# Oxidative stress accelerates repeat sequence instability and base substitutions promoting gastrointestinal driver mutations in MSH2 deficient mice

**DOI:** 10.1186/s41021-025-00342-y

**Published:** 2025-10-23

**Authors:** Mizuki Ohno, Noriko Takano, Kyoko Hidaka, Fumiko Sasaki, Yasunobu Aoki, Takehiko Nohmi, Teruhisa Tsuzuki

**Affiliations:** 1 Department of Medical Biophysics and Radiation Biology, Faculty of Medical Sciences, Kyushu University, 3-1-1 Maidashi, Higashi-Ku, Fukuoka, 812-8582 Japan; 2 Center for Fundamental Education, University of Kitakyushu, 4-2-1 Kitagata, Kokuraminami-Ku, Kitakyushu, Fukuoka 802-8577 Japan; 3 Health and Environmental Risk Division, National Institute for Environmental Studies, 16-2 Onogawa, Tsukuba, Ibaraki 305-8506 Japan; 4 Division of Genetics and Mutagenesis, National Institute of Health Sciences, 3-25-26 Tonomachi, Kawasaki-Ku, Kawasaki, Kanagawa 210-9501 Japan; 5 Department of Comprehensive Oncology, Faculty of Medical Sciences, Kyushu University, 3-1-1 Maidashi, Higashi-ku, Fukuoka, 812-8582 Japan; 6 Department of Science and Engineering, Yamato University, 2-5-1 Katayama-Cho, Suita-Shi, Osaka, 564-0082 Japan; 7 Professor Emeritus, Faculty of Medicine, Kyushu University, 3-1-1 Maidashi, Higashi-ku, Fukuoka, 812-8582 Japan

**Keywords:** Mismatch repair, Oxidative stress, Potassium bromate, Microsatellite Instability, Transgenic rodent assay, Mutagenesis, Tumorigenesis

## Abstract

**Background:**

Loss of DNA mismatch repair (MMR) increases mutagenesis and tumorigenesis. mutS homolog 2 (MSH2), a central component of the MMR pathway, is essential for correcting base–base mismatches and insertion/deletion loops during DNA replication. To investigate how *Msh2* deficiency cooperates with oxidative stress to drive mutagenesis and tumorigenesis, we employed an *rpsL* reporter gene assay using normal tissues before tumor development following treatment with an oxidizing agent.

**Results:**

The background mutation frequency in the small intestines of *Msh2*^*-/-*^ mice was over 20-fold higher than that of wild-type mice. In addition to G > A base substitutions, frequent 1-bp deletions in adenine mononucleotide repeats ((A)n) in the *rpsL* gene were observed. Potassium bromate treatment further increased the mutation frequency, particularly insertion-deletion mutations (indel), in the normal small intestinal epithelium of *Msh2*^*-/-*^ mice before tumor development. Mutation signature analysis from next-generation sequencing data revealed that signatures associated with MMR deficiency (SBS15, SBS44, and ID2) and clock-like processes (SBS1 and SBS5) were consistently detected across all *Msh2*^*-/-*^ tumors, similar to those observed in human MMR-deficient cancers. ID2, which involves 1-base deletions occurring in (A/T)_n_ tracts of six bases or longer, supports the findings of the *rpsL* assay. Microsatellite instability (MSI) analysis showed that indel mutations at (A)n loci detected using the *rpsL* assay reflect genome-wide MSI. *Msh2*^*-/-*^ tumors frequently harbored driver mutations, such as frameshift mutations in short tandem repeats within *Apc* and G > A substitutions in *Ctnnb1*, both of which activate the Wnt signaling pathway. Oxidative stress further accelerated these mutational processes.

**Conclusion:**

Oxidative stress promotes repeat-associated mutagenesis, which manifests as MSI and base substitutions in MMR-deficient intestinal tissues, thereby enhancing the mutator phenotype and increasing the overall mutation burden. This process can be sensitively captured using our *rpsL* assay, which serves as a functional indicator of MMR deficiency and replication instability in normal tissues before tumor formation. This increases the likelihood of driver mutations in oncogenes and tumor suppressor genes, ultimately accelerating early tumorigenesis. This study demonstrated that MSH2 is essential for maintaining genome stability under oxidative conditions and functions as a key suppressor of oxidative stress–induced tumorigenesis.

**Supplementary Information:**

The online version contains supplementary material available at 10.1186/s41021-025-00342-y.

## Introduction

Cancer is a genetic disease that originates from somatic mutations occurring in normal cells and drives cellular phenotypes such as uncontrolled growth and survival [1]. These mutations arise from DNA replication errors and from incomplete or defective DNA damage repair [2]. The gastrointestinal tract exhibits a higher incidence of tumorigenesis than other organs, which may be attributed to the accumulation of replication errors due to its persistently high proliferative activity even in adulthood, as well as an increased level of DNA damage-induced mutagenesis [3–5].

Oxidative stress, induced by various exogenous and endogenous stimuli, promotes the intracellular generation of reactive oxygen species, which in turn constitute a major source of spontaneous DNA damage [6, 7]. Oxidative DNA lesions are normally repaired by appropriate DNA repair pathways; however, if they remain unrepaired or are improperly repaired, they can result in mutations [8]. These mutations may initiate events during tumorigenesis. This is supported by the fact that loss-of-function mutations in *MUTYH* and *NTHL1*, which encode base-excision repair (BER) enzymes responsible for repairing oxidatively damaged bases, have been recognized as genetic causes of hereditary colorectal cancer syndromes [9–11]. Our previous study has demonstrated that administration of potassium bromate (KBrO₃), an oxidizing agent, to *Mutyh*-deficient mice resulted in a several-fold increase in the number of intestinal tumors [12]. Furthermore, exposure to KBrO₃ markedly increased G:C > T:A transversions in normal small intestinal tissues before tumor development. This type of mutation is strongly associated with tumor burden and is frequently detected in oncogenic hotspot codons of *Apc* and *Ctnnb1* [12, 13]. This suggests that mutation types shaped by both mutagen exposure and repair deficiency are more predictive of oncogenic risk than overall mutation frequency.

DNA mismatch repair (MMR) plays a key role in preventing tumor development. MMR suppresses the mutations generated by replication errors and DNA damage-induced mismatched bases [14]. Pathogenic mutations in MMR genes have been identified as the cause of Lynch syndrome, an autosomal dominant hereditary cancer predisposition syndrome characterized by the development of colorectal and various other organ cancers [11, 15, 16]. mutS homolog 2 (MSH2) is an essential component of the MMR function, which recognizes and initiates the repair of base–base mismatches and small loops that arise during DNA replication [17]. MSH2 forms heterodimers with either MSH6 to detect single-base mismatches and small loops or with MSH3 to recognize larger loops. MSH2 deficiency impairs MMR, resulting in base substitution and insertion/deletion (indel) mutation at simple tandem repeat sequences (hereafter referred to as microsatellite (MS)) [14, 17, 18]. Similar to MUTYH deficiency, the loss of MSH2 likely does not provide an immediate proliferative advantage but rather contributes to tumorigenesis by establishing a mutator phenotype that promotes the accumulation of mutations in cancer-relevant genes.

*Msh2*^*-/-*^ mice, previously established in our laboratory [19], spontaneously develop intestinal tumors, and the frequency of tumor formation was elevated more than 20-fold by oral administration of KBrO₃ for 16 weeks [20]. Pathogenic missense mutations in *Ctnnb1* were identified in tumor cells [20, 21]. However, as assessed by the *gpt*-delta reporter gene assay, we did not observe a significant increase in the overall mutation frequency in the normal small intestinal tissues of *Msh2*^*-/-*^ mice following KBrO₃ administration, although characteristic patterns of base substitutions and indels associated with MMR deficiency were observed [20, 21].

To better understand how oxidative stress interacts with the MMR pathway to influence mutagenesis and tumorigenesis, we employed a multifaceted approach combining the *rpsL* reporter gene assay [12, 22] for the highly sensitive detection of somatic mutations, genome-wide profiling of mutation patterns in tumor samples using next-generation sequencing (NGS), and microsatellite instability (MSI) analysis. In this study, we demonstrate that oxidative stress induced a distinct mutational profile in MMR-deficient tissues, characterized by increased base substitutions and a striking accumulation of indels within adenine mononucleotide tracts, as revealed by the *rpsL* reporter assay. These indel patterns may serve as predictive markers for genome-wide MSI.

## Materials and methods

### Mice and tissue sample collection

**Fig. 1:**
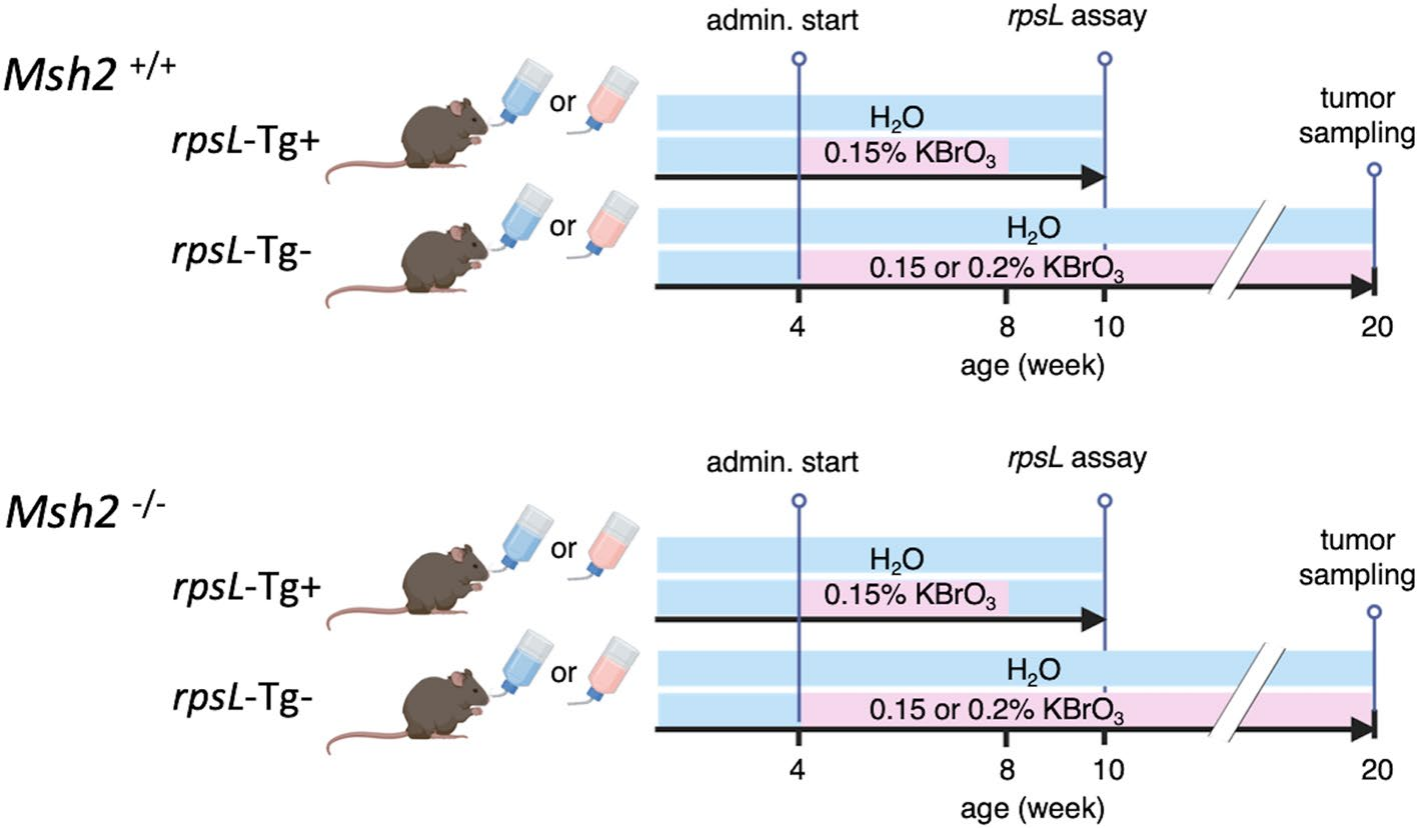
Experimental design for KBrO₃ administration. Both *Msh2*^*+/+*^ and *Msh2*^*-/-*^ mice were divided into two groups: a control group receiving regular drinking water (blue) and a treatment group receiving KBrO₃ (pink). For the *rpsL* assay, small intestine samples from *rpsL* transgenic mice (*rpsL*-Tg +) were collected two weeks after switching to water following a 4-week 0.15% KBrO₃ administration. Tumor samples were collected from the small intestine of mice treated with 0.15% or 0.2% KBrO₃ for 16 weeks

The *Msh2* gene knockout mouse line was established previously in our lab [20] and maintained by backcrossing with C57BL/6Jc mice (CLEA Japan, Inc., Tokyo, Japan). For the in vivo somatic mutation assay, *rpsL* transgenic (Tg) mice [19, 22] were used. *Msh2*^*+/−*^female and *Msh2*^*+/−*^/*rpsL*-Tg male mice were mated to produce *Msh2*^*-/-*^ /*rpsL*-Tg and *Msh2*^*+/+*^ /*rpsL*-Tg mice. For the *rpsL* mutation assay, mice were orally administered either regular water or 0.15% KBrO₃-containing water ad libitum for four weeks starting at 4 weeks of age, followed by regular water for an additional two weeks (Fig. 1). Tissue samples were collected at 10 weeks of age. For tumor analysis, mice were continuously treated with 0.15% KBrO₃-containing water from 4 weeks of age for 16 weeks, and tumors along with normal small intestinal tissues were collected at 20 weeks of age (Fig. 1). Mice were sacrificed after completion of the treatment schedule, and tissue samples were dissected. For the *rpsL* mutation assay, tissues were immediately frozen in liquid nitrogen and stored in a − 80 °C deep freeze. For NGS genome analysis, the isolated organs were put into RNAlater (Thermo Fisher Scientific Inc., Massachusetts, USA) and stored in a − 30 °C freezer. All animal care and procedures were approved by the Institutional Animal Care and Use Committee of Kyushu University (approval nos. A22-027, A20-089, A30-147, and A28-112). All experiments were performed in accordance with the Guidelines for the Proper Conduct of Animal Experiments of the Science Council of Japan.

### Mutation assay using rpsL transgenic mice

The small intestines were isolated by cutting the stomach at the upper duodenum and the end of the ileum. They were divided into three parts, and the part containing the upper duodenum was used for the *rpsL* assay. The *rpsL* mutation assay was performed as previously described [12]. Briefly, extracted genomic DNA was digested with Ban II (Takara Bio Inc.) at 37 °C for 2 h. The *rpsL* reporter gene containing 3 kb fragments of pSSW [19, 22] was self-ligated using T4 ligase (Takara Bio Inc.) to generate circular plasmids. *Escherichia coli* 10-beta strain (cat. C3020, New England Biolabs, Ipswich, MA, USA), a derivative of DH10B, which is kanamycin (Km)-sensitive and streptomycin (Sm)-sensitive, was transformed using self-ligated shuttle vectors. Transformation efficiency was monitored based on the number of Km-resistant colonies. Mutations in the *rpsL* coding region that confer Sm resistance to host bacteria can be selectively detected. The overall mutation frequency (MF) was calculated by dividing the number of Km/Sm-resistant colonies by the estimated total number of Km-resistant colonies. To detect mutations in the *rpsL* coding region, Sanger sequencing was performed on every Km/Sm-resistant colony after colony PCR. The MF of each mutation type was calculated based on the ratio of the corresponding mutation type to the total number of mutations.

### Genomic sequencing analysis

DNA extracted from small intestinal tumors, normal regions of small intestines, and heart tissues of KBrO_3_-treated *Msh2*^*-/-*^ mice were subjected to whole-exome sequencing (WES) according to the previously described method [12], with some modifications. Briefly, the extracted DNA was used for target enrichment and library preparation using the SureSelectXT Mouse All Exon Kit (Agilent Technologies, Santa Clara, CA, USA) and sequenced on the Hiseq2000 platform (Illumina, San Diego, CA, USA) with 100-bp paired ends or on the Novaseq 6000 platform (Illumina) with 150-bp paired ends. Sequenced reads were mapped to the mouse reference genome (GRCm38/mm10) using BWA-MEM v. 0.7.17 [23]. For whole-genome sequencing (WGS), a library was prepared using the TruSeq DNA PCR-free kit (Illumina), and a 150-bp paired-end sequence was obtained using NovaSeq X Plus (Illumina).

### Detection of tumor-specific somatic mutation and analysis of mutation pattern

After applying base quality score recalibration, somatic mutations were detected using GATK Mutect2 v.4.2.0.0 [24] in tumor-normal mode, using the heart or normal regions of the small intestine as the normal reference. Based on the criteria described by Lange et al. [25], we retained variants only if the total read depth was ≥ 10 in both tumor and normal samples, with ≥ 3 variant-supporting reads in the tumor and none in the normal. Germline resources were not used owing to their unavailability. Variants located on chromosomes X, Y, and M or present in the dbSNP were excluded. Mutations shared by two or more tumors were removed. Finally, all the remaining variants were annotated using SnpEff v.5.1. The scripts used in the analysis are provided in the Supplemental Code.

Mutation patterns of SNV and indels were analyzed using the COSMIC Sigprofiler MatrixGenerator (https://github.com/AlexandrovLab/SigProfilerMatrixGenerator) and SigProfilerExtractor (https://github.com/AlexandrovLab/SigProfilerExtractor). Mutational signature data (mm10, Mus musculus version 3.3, Human SBS and ID signature ver3.4) were downloaded from COSMIC (https://cancer.sanger.ac.uk/signatures/downloads/).

### MSI analysis

MSI analysis was performed using MSIsensor-pro (v1.2.0) [https://github.com/xjtu-omics/msisensor-pro]. The pipeline consisted of three steps: (1) S regions were identified from the reference genome using the scan module; (2) a tumor-only detection baseline was constructed using the baseline module based on WES data from 29 and WGS data from 22 normal tissue samples expected to be microsatellite stable, including the samples form C57BL/6Jc wild-type mice and MMR-proficient mice with mutations unrelated to MMR; and (3) MSI scores were computed for tumor-only samples using the pro module. Most normal samples were sequenced in-house, with a minority sourced from publicly available datasets. For each microsatellite site listed in the.dis file output by MSIsensor, Shannon entropy (SE) was calculated to quantify allelic diversity, which serves as an indicator of the stability status of each microsatellite site. Higher values indicate greater diversity. The mean SE values were calculated and stratified by motif and repeat length for each sample. To compare the relationship between SE and repeat length across samples, data were grouped by motifs. Data from complementary sequences were combined, and the corresponding mean SE values and total site counts were summarized. MS loci with site counts of < 10 in the WES were excluded from the analysis.

### Fragment analysis

Genomic DNA was isolated from normal tissue or tumors of the small intestines of wild-type and *Msh2*^*-/-*^ mice. Fragment analysis of the microsatellite loci was performed by SystemBiotics Corporation (Kanagawa, Japan). Genomic DNA was PCR-amplified using fluorescently labeled primers for each target MS locus (D3mit10 and mBat37), and fragment separation was performed using capillary electrophoresis with an Applied Biosystems 3130xl Genetic Analyzer (Thermo Fisher Scientific). Allele sizing and peak calling were conducted using the GeneMapper software.

### Detection of the rpsL integrated genomic region

Genome DNA of *Msh2*^*-/-*^*/rpsL* mice was subjected to WGS. To identify the genomic region in which the *rpsL*-containing plasmid pSSW was integrated, chimeric reads containing both pSSW and mouse genomic sequences were extracted and analyzed. First, paired-end reads were selected, in which one read was mapped to the mouse genome (mm10), but its mate was unmapped. The unmapped mates were re-aligned using the pSSW reference sequence. From the resulting alignments, read pairs where one mate mapped to pSSW and the other remained unmapped were extracted and re-aligned to the mouse genome. The insertion site was estimated based on clustering of the re-aligned reads and visualized using IGV. The detailed pipeline used to extract and realign the chimeric reads is provided in the Supplemental Code.

## Results

### Profiling of somatic mutations in normal tissues preceding tumorigenesis using the rpsL assay

To investigate the impact of oxidative stress on mutagenicity in normal intestinal tissues under mismatch repair proficient or deficient conditions, we subjected *Msh2*^*+/+*^ and *Msh2*^*-/-*^ mice to chronic exposure to oxidative stress by providing 0.15% of KBrO_3_-containing water for four weeks (Fig. 1). Somatic mutation analysis was performed using normal mucosal tissue from the small intestines of *rpsL* transgenic mice. We found that multiple tandem repeat units of the pSSW shuttle vector containing the *rpsL* coding sequence were inserted at a single locus within the 111.7 Mb region of chromosome 7 in this mouse line.

**Fig. 2:**
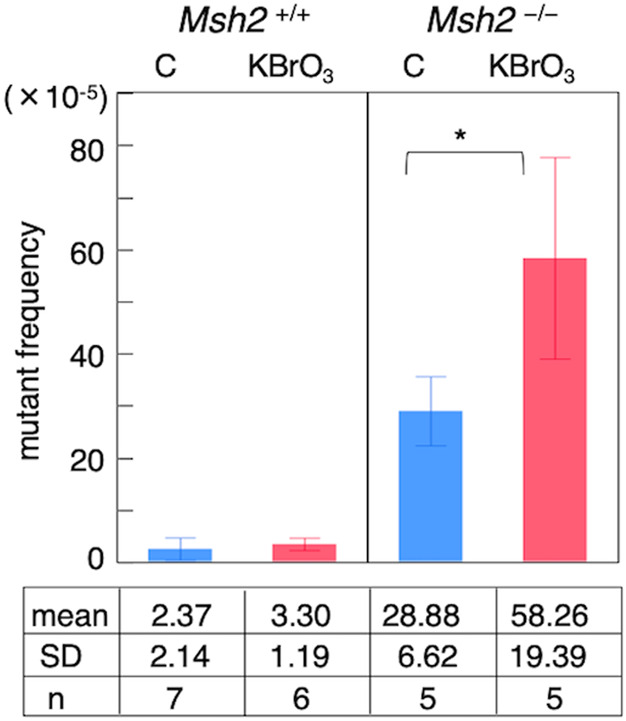
Mutant frequency in small intestines of *Msh2*^*+/+*^ and *Msh2*^*−/−*^ mice. The mean mutant frequency ± SD is shown. The number of mice used in each group (*n*) and the corresponding mean values are shown in each column. C: control; KBrO₃: 0.15% potassium bromate–treated. *p < 0.05, Mann–Whitney U-test

Mutant frequency was calculated by dividing the number of Km/Sm-resistant mutant colonies by the total number of Km-resistant colonies screened. Mean mutant frequency in control *Msh2*^*+/+*^ mice was 2.37 × 10^-5^, in control *Msh2*^*-/-*^ mice was 28.88 × 10^-5^, which was more than 20 times higher than that of wild-type mice (Fig. 2). KBrO₃ exposure elevated the mutant frequency to 58.26 × 10^-5^, approximately a twofold increase compared with that in the control group of *Msh2*^*-/-*^ mice (p < 0.05, Fig. 2). In wild-type mice, an increase was observed following KBrO₃ exposure; however, it was not statistically significant. Next, we calculated the MF using the spectra based on the relative proportion of each mutation type within the total mutant frequency for each mouse (Table 1).

**Table 1:** Mutation frequency

	*Msh2* ^+/+^		*Msh2* ^−/−^	
MF (✕10^–5^)	Control	KBrO_3_ (0.15%)	Control	KBrO_3_ (0.15%)
BS total	0.37	1.0	4.82	4.22
G:C > A:T	0.30	0.53	2.64	2.39
A:T > G:C	-	0.18	0.94	1.51
G:C > T:A	0.04	0.29	0.37	0.21
G:C > C:G	-	-	0.16	0.11
A:T > T:A	0.03	-	0.22	-
A:T > C:G	-	-	0.49	-
Indel total	2.0	2.32	24.79	54.97*
1-indel	0.71	1.38	1.18	0.81
1-indel_(A)_n_	0.06	0.11	22.82	52.69*
2-indel_(A)_n_	-	-	0.21	-
> 2bp_indel	1.23	0.83	0.58	1.47
other	-	0.07	-	-

- not detected, * *p* < 0.05, Mann-Whiteny U-test

In *Msh2*^*+/+*^ mice, the MF of base substitution (BS) and indel were 0.37 × 10^-5^ and 2.0 × 10^-5^ in the control group and 1.0 × 10^-5^ and 2.32 × 10^-5^ in the KBrO_3_-treated group, respectively. In *Msh2*^*-/-*^ mice, MF of BS in the control and KBrO_3_-treated groups were 4.82 × 10^-5^ and 4.22 × 10^-5^, respectively, with approximately 75% comprised of G:C > A:T and A:T > G:C transitions. KBrO_3_ exposure increased the A:T > G:C frequency. A high indel MF of 24.79 × 10^-5^ was observed even in control mice, corresponding to an indel/BS ratio of approximately 5:1. Most indels consisted of single-base deletions in the (A)n mononucleotide repeat tract. KBrO₃ exposure significantly increased the frequency of these mutations (Mann–Whitney U-test, p < 0.05), primarily due to a surge in 1-bp deletions at adenine repeat sites of lengths (A)₂_–_₆, which served as mutation hotspots in the *rpsL* gene (Fig. S1). The adenine indel MF was positively correlated with repeat length, with the (A)₆ site, the longest in *rpsL*, showing the highest MF. Importantly, KBrO₃ treatment further enhanced MF at (A)_n_ sites (Table 2).

**Table 2:** MF of adenine 1-indel by repeat length in *rpsL* gene region

	KBrO_3_	
sites	0%	0.15%
(A)_2_	0.07	0.21
(A)_3_	0.23	0.56
(A)_4_	0.02	0.32
(A)_5_	4.93	10.68
(A)_6_	19.20	45.94

KBrO₃ tended to exert a stronger effect on indel frequency at longer mononucleotide repeats. These results suggest that exposure to oxidative stress impairs accurate DNA replication in repetitive sequences, particularly under MMR-deficient conditions. The characteristic indel patterns observed in *rpsL* reporter genes reflect genome-wide MSI.

### Tumor-specific somatic mutations detected using NGS data

**Fig. 3:**
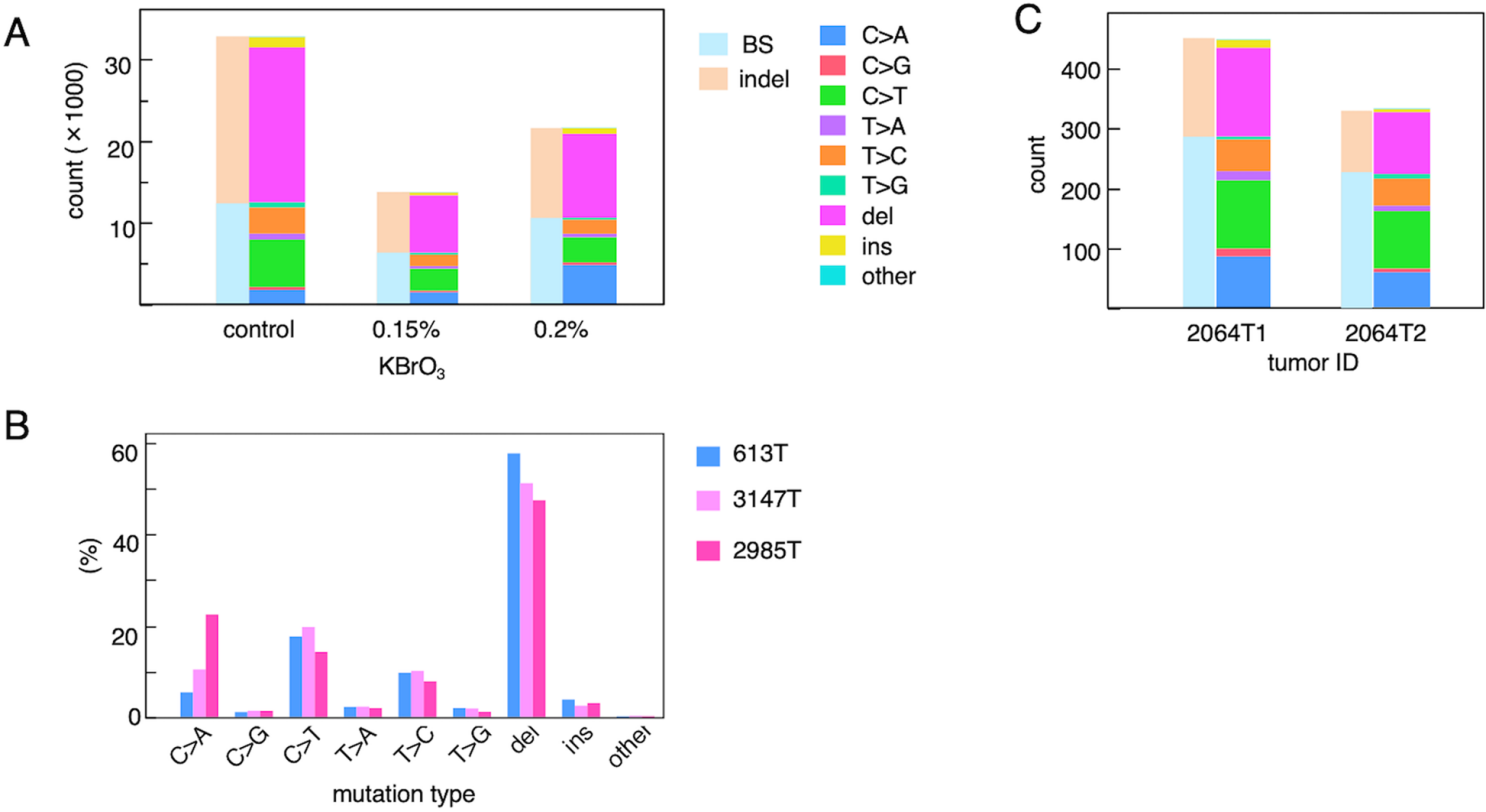
Somatic mutations in *Msh2*^*−/−*^ tumors detected using WGS and WES. Tumor-specific mutations were identified using WGS or WES of genomic DNA isolated from small intestinal tumors derived from either untreated control mice (spontaneously developed tumors) or mice treated with 0.15% or 0.2% KBrO_3_. **A** Mutation burden in individual tumors categorized by mutation type using WGS data. The stacked bar chart shows the absolute number of mutations in each tumor grouped into single-base substitution (BS) and indel classes (left). The BS were further classified into 6-substitution types (right). The y-axis indicates absolute counts. **B** Relative mutation spectra of each tumor shown in (**A**). Each bar represents the proportion of each mutation type normalized to the total number of mutations in the tumor. The y-axis indicates percentage of each component. **C** The stacked bar chart shows the mutation burden in individual tumors based on WES data from two tumors derived from mice treated with 0.15% KBrO_3_. Mutation classification is shown in (**A**). The y-axis indicates absolute counts

To investigate whether the mutation pattern observed in *Msh2*^*-/-*^ mice using the *rpsL* assay is also present in the mouse genome and to explore how oxidative stress levels may influence the mutation profile, we conducted WES and WGS on tumor samples and matched normal tissue samples (Fig. 1). No tumor samples from wild-type mice were available for analysis, as tumors rarely develop either spontaneously or after KBrO₃ treatment. Tumor-specific somatic mutations were identified using the Mutect2 tumor-normal model. Mutational signature analyses, including single-base substitution (SBS) and insertion-deletion (ID) signatures, classify somatic mutation patterns observed in human cancers into distinct profiles associated with specific mutagenic processes, as previously described by Alexandrov et al. [26]. Using this approach, we analyzed the variant data using a basic classification framework. It should be noted that, in accordance with the convention used in this analysis, base substitutions are described using the pyrimidine base of the mutated Watson–Crick pair. We observed a mutation profile similar to that detected in the *rpsL* assay, which was characterized by a high frequency of indels and a comparable BS spectrum (Fig. 3). Deletions occurred more frequently than insertions. In the WGS results, C > T was the most prevalent among the BS, accounting for over 40% of the total, followed by T > C and C > A (Fig. 3A). In the WES results (Fig. 3C), a similar base substitution spectrum was observed. Compared with WGS, WES showed a lower proportion of indels, likely because exonic regions contain fewer repetitive sequences, which reduces the relative contribution of indels to the overall mutation profile. The proportion of C > A transversions, which are commonly associated with oxidative stress, was higher in tumors from mice treated with 0.15% KBrO₃ than in spontaneous tumors and further increased in those treated with 0.2% KBrO₃ (Fig. 3B). These findings suggested that DNA damage induced by the oxidizing agent increased in a dose-dependent manner.

**Fig. 4:**
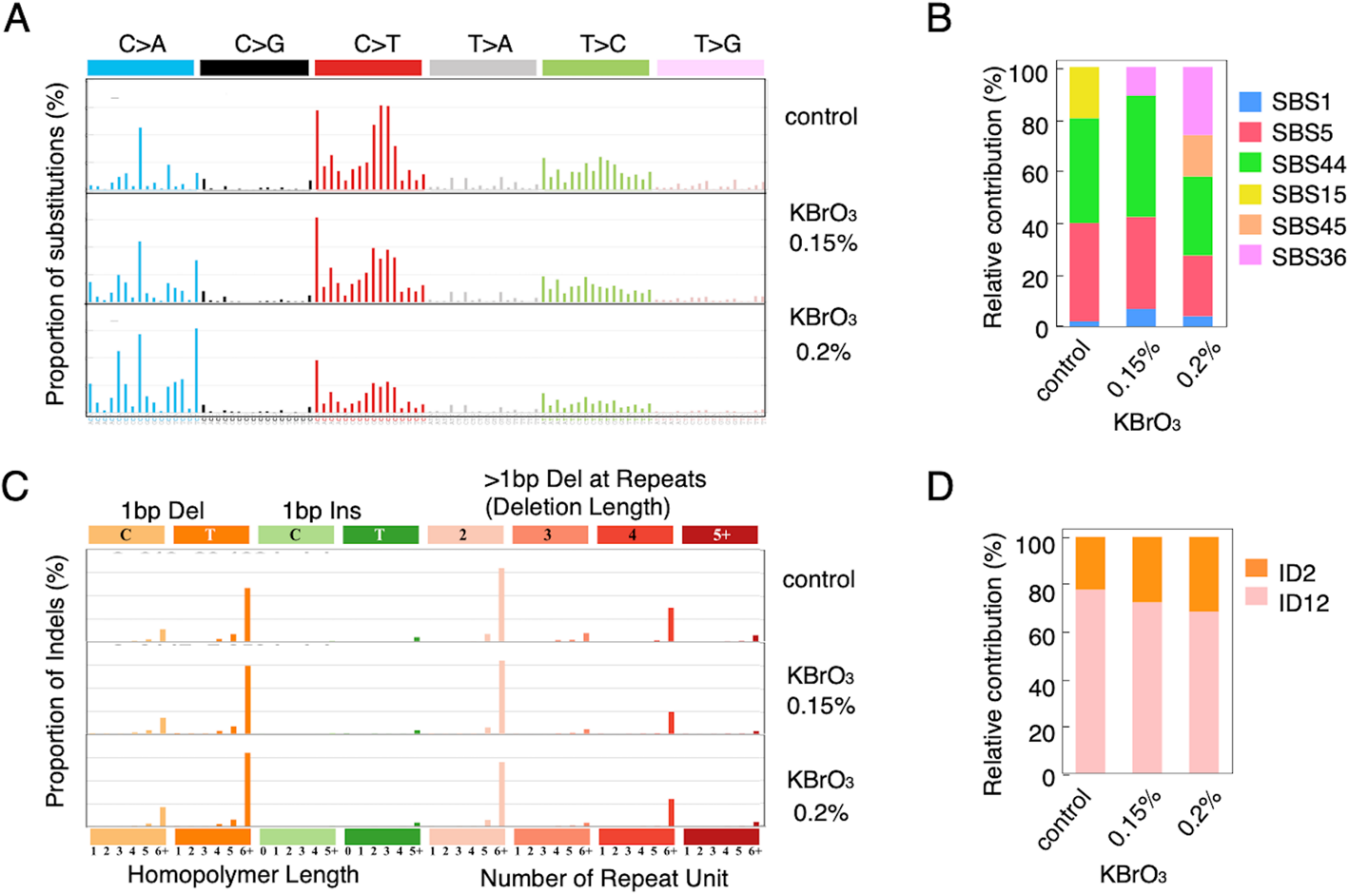
Mutational profiles in *Msh2*^*−/−*^ tumors. Mutational profiles based on tumor-specific variants identified in WGS of tumors derived from either control mice or mice treated with 0.15% or 0.2% KBrO₃. **A** A one-base substitution mutation pattern is displayed according to the 96-base substitution classification defined by the substitution class and sequence context immediately 3′ and 5′ to the mutated base. The y-axis indicates the percentage of each mutation relative to the total number of mutations. The x-axis indicates the 96 trinucleotide types. **B** Relative contributions of SBS mutational signatures corresponding to the data shown in Figure **A**. **C** Indel patterns. The height of each bar in the mutational profile represents the proportion of one indel mutation type among all the indel mutation types in the signature. For example, the tallest orange bars indicate 1-bp deletions occurring in T homopolymers longer than 6 bp, and the tallest pink bars indicate deletions larger than 1 bp in repeat sequences with more than six repeat units. **D** Relative contributions of ID signatures corresponding to the data shown in Figure **C**

The SBS profiles derived from the WGS and WES data showed highly similar patterns (Fig. 4A, Fig. S2), with consistent enrichment of C > A and C > T mutations, supporting the robustness of the mutational signature associated with oxidative stress and mismatch repair deficiency. The SBS patterns of WGS fell into three distinct categories (Fig. 4A and B): (1) aging-associated clock-like signatures, such as SBS5 and SBS1; (2) MMR deficiency-associated signatures, such as SBS15 or SBS44, which are known to be found alongside frequent indels at repeat sequences, including ID2; and (3) SBS36, a signature attributed to oxidative DNA damage involving 8-oxoguanine. Signatures from the first and second categories were consistently detected across all three samples, regardless of KBrO_3_ exposure. By contrast, SBS36 was specifically observed in the KBrO_3_-treated samples, with a higher contribution in the 0.2% treatment than in the 0.15% treatment.

The indel profile was characterized using ID signatures (Fig. 4C, B, Fig. S2). A high frequency of 1-dels longer than 6 bp was observed at thymine homopolymer sites (complementary to adenine), corresponding to the ID2 signature. ID2 exhibits a markedly increased mutation burden in human cancer samples with MSI-high status. Additionally, analysis of the WGS data revealed frequent deletions of > 1 bp, a hallmark of the ID12 signature, which we thought reflected deletions of one or a few repeat units in di or trinucleotide repeat sequences. The ratio of 1-del (T)_>6_ to > 1-bp deletions increased from untreated to 0.15% and further to 0.2% KBrO₃-treated mice. Although 1-bp deletions at (C)_>6_ were observed less frequently, they also exhibited a dose-dependent increase with KBrO₃ exposure. In contrast, this ID12-associated pattern was not observed in WES data. This may be attributed to differences in the distribution of di- and trinucleotide repeat sequences between the coding and intergenic regions (Fig. S2B).

### MSI analysis

**Fig. 5:**
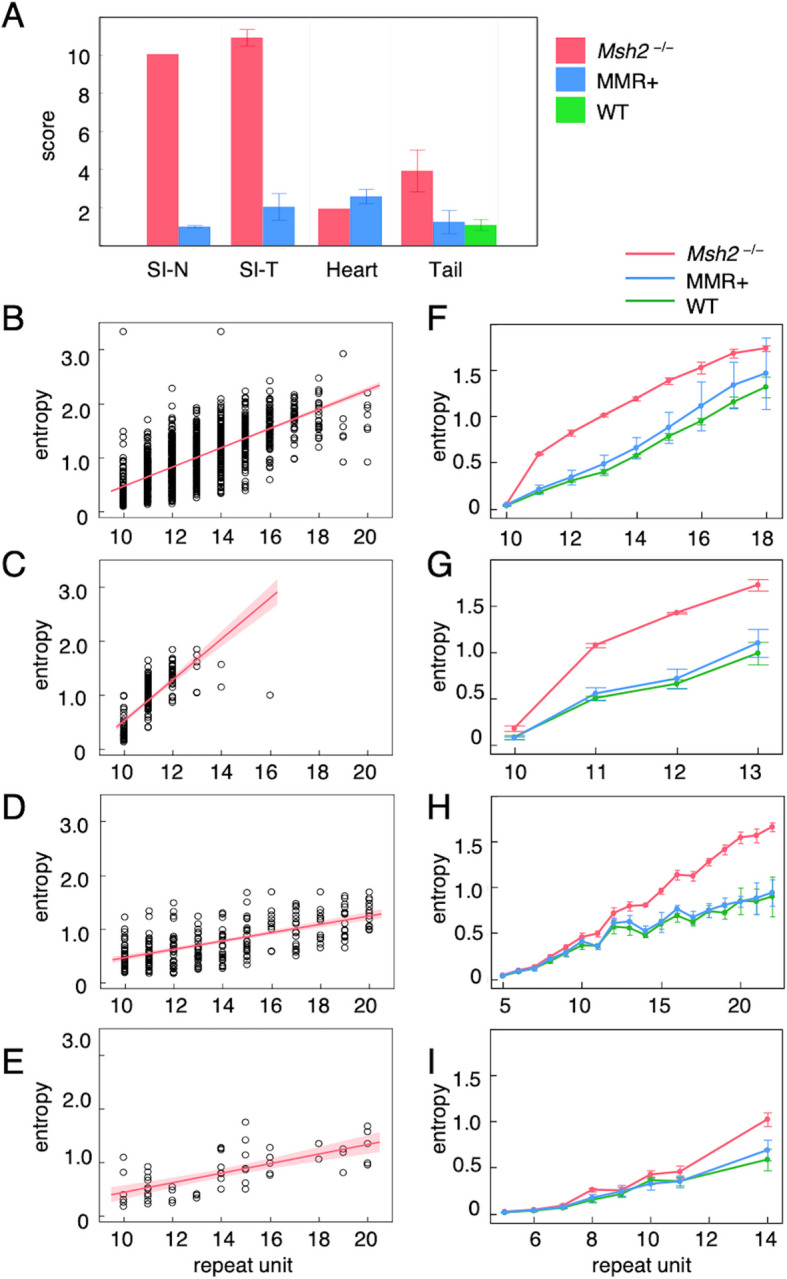
Comparative analysis of repeat length variation across genotypes, tissue types, and motif classes. (**A**) MSI scores (mean ± SD) across different tissue types and genotypes. Small intestinal normal tissues (SI–N), small intestinal tumors (SI-T), and heart tissues were collected from *Msh2*^*–/–*^ mice (magenta) and MMR-proficient *Mutyh*^–/–^ mice (blue), both treated with 0.15% KBrO_3_ for 16 weeks. Tail tissues were obtained from untreated *Msh2*^*–/–*^, *Mutyh*^–/–^, and wild-type (WT, green) mice. Elevated MSI scores are observed specifically in intestinal tissues from *Msh2*^*–/–*^ mice. (B–E, left) Shannon entropy (SE) values for individual repeat sites plotted against repeat unit length for different motif types in small intestinal tumors from KBrO_3_-treated *Msh2*^*–/–*^ mice. SE reflects the degree of repeat length variation: SE = 0 indicates complete stability where all reads share the same repeat length, whereas higher values indicate coexistence of multiple repeat lengths, representing greater instability. Each dot represents the SE of a single repeat site. The magenta lines indicate linear fits, and the shaded areas represent confidence intervals. These scatterplots highlight motifs in which SE values increase with repeat length, showing that loci with longer repeats tend to display greater instability. Each motif is shown as (**B**) A/T, (**C**) G/C, (**D**) CA/TG, and (**E**) GA/TC. (**F**–**I**, right) Average SE (mean ± SD) plotted against the repeat unit length for each motif type. Data points represent the means across samples and are connected by linear lines for visualization. Magenta: Small intestinal tumors (n = 2) and normal tissues (*n* = 1) from KBrO_3_-treated *Msh2*^*–/–*^ mice. Blue: Small intestinal tumors (*n* = 4) and normal tissues (*n* = 2) from KBrO_3_-treated *Mutyh*^–/–^. Green: Tail tissues from untreated wild-type mice (*n* = 5). Each motif is shown as (**F**) A/T, (**G**) G/C, (**H**) CA/TG, and (**I**) GA/TC. These plots demonstrate that *Msh2*^*–/–*^ mice exhibit length-dependent increases in SE, most prominently at A/T and CA/TG motifs, showing greater instability compared with both untreated and KBrO₃-treated MMR-proficient mice

To further investigate repeat length variations at simple repeat sites beyond adenine or complementary thymine base (A/T) homopolymers, we focused our analysis on MS regions across the genome. First, we evaluated the MSI score, which was defined as the proportion of unstable sites among the MS loci. WES data from KBrO₃-treated *Mutyh*⁻^/^⁻ mice were included as MMR-proficient controls subjected to the same oxidative stress. The mean MSI scores varied according to genotype and organ type. The tail samples from untreated wild-type mice and organs from KBrO₃-treated MMR-proficient mice were remarkably low, indicating that MS regions were largely stable (Fig. 5A). In KBrO_3_-treated *Msh2*^*−/−*^ mice, the intestinal tissues exhibited the highest scores, regardless of whether they were normal or tumor tissues, indicating a strong susceptibility to MSI in this organ. The samples from heart and tails, which were less directly affected by oral administration of KBrO₃, showed significantly lower scores compared with those of intestinal samples (Fig. 5A). No significant difference in MSI scores was observed between control or KBrO₃-treated *Msh2*^*−/−*^ mice in either tissue (data not shown).

It is well established that the extent of microsatellite instability varies greatly depending on the repeat motif and unit length. Therefore, it is not straightforward to directly compare instability across different motifs and repeat lengths throughout the genome. To address this challenge and to identify microsatellite sites that are particularly prone to instability in the absence of MSH2, we devised and applied a novel approach. Specifically, we assessed the variant read status at each microsatellite site, stratified by motif and repeat length, using Shannon entropy (SE). SE provides a quantitative measure of diversity in read composition at a given locus. In this context, SE reflects variation in repeat length: a value of zero indicates complete stability, where all reads share the same repeat length, whereas higher values indicate the coexistence of multiple repeat lengths, representing greater instability.

We identified several motifs, such as A/T, C/G, and CA/TG, whose SE increased in accordance with the number of repeat units in tumor samples from KBrO₃-treated *Msh2*^*–/–*^ mice (Fig. 5B–E). These plots highlight the motifs that exhibit a positive correlation between repeat length and SE values, indicating that loci with longer repeat units tend to show higher SE and thus greater instability. In general, microsatellite instability showed a positive correlation with repeat length, a finding that recapitulates a well-established hallmark of MSI. Among the mono- and dinucleotide repeat motifs showing a positive correlation between SE and the number of repeat units, A/T, C/G, and CA/TG exhibited significantly higher rates of increase than those observed in wild-type and KBrO₃-treated MMR-proficient mice samples which enabled comparison across genotypes (Fig. 5F–I). In the case of GA/TC and AG/CT motifs, SE were higher in *Msh2*^*–/–*^ mice only at sites with longer repeat units. Collectively, these results indicate that loss of MSH2 leads to length-dependent destabilization of specific repeat motifs, most prominently A/T and CA/TG repeats in intestinal tissues exposed to oxidative stress.

**Fig. 6:**
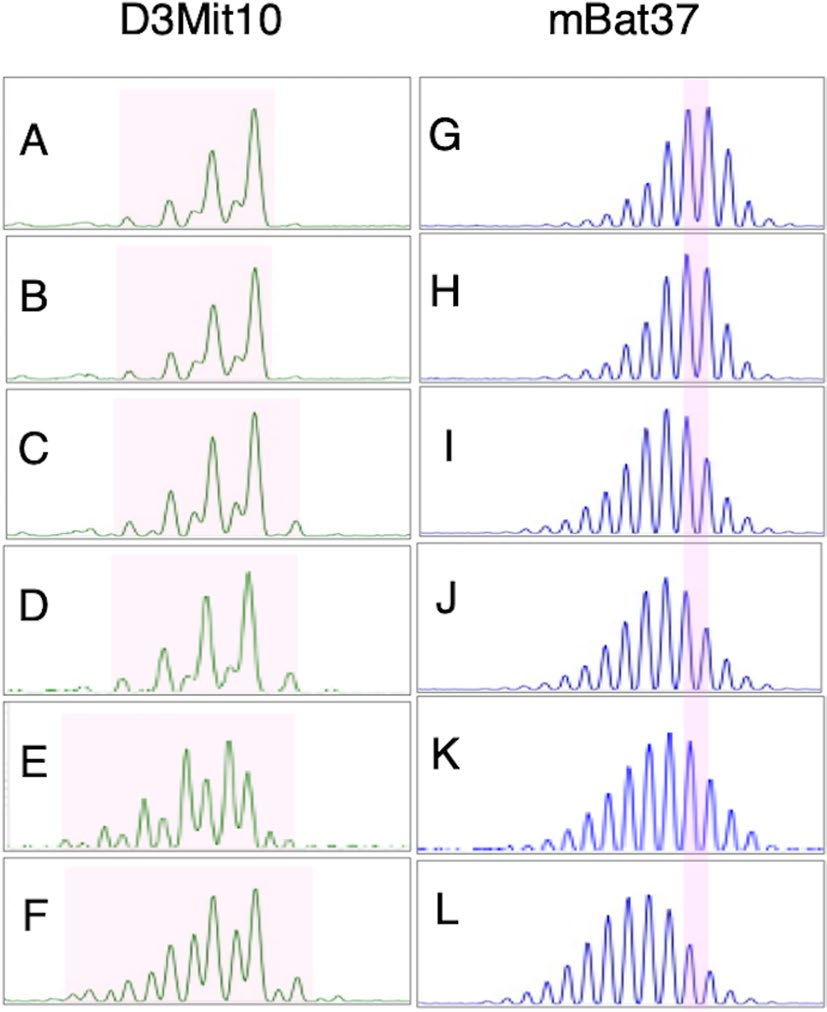
Fragment analysis. Fragment size distributions obtained from PCR-based MSI analysis. All samples are derived from normal small-intestinal tissues. (**A**–**F**) D3Mit10 (TG dinucleotide repeat, *n* = 26); (**G**–**L**) mBat37 (adenine mononucleotide repeat, *n* = 37). (**A**, **G**) 10-week-old control wild-type; (**B**, **H**) 10-week-old wild-type treated with KBrO₃ for 4 weeks; (**C**, **I**) 10-week-old control *Msh2*^*–/–*^; (**D**, **J**) 10-week-old *Msh2*^*–/–*^ treated with KBrO₃ for 4 weeks; (**E**, **K**) 20-week-old control *Msh2*^*–/–*^; (**F**, **L**) 20-week-old *Msh2*^*–/–*^ treated with KBrO₃ for 16 weeks. In D3Mit10, the positions and relative heights of the three major peaks are generally consistent in **A-D**. In contrast, in the 20-week-old mice **E** and **F**, both untreated and KBrO₃-treated, multiple peaks emerge and the total peak width (highlighted in pink) increases, indicative of MSI-high status. For mBat37, comparison of the untreated 10-week-old *Msh2*^*–/–*^ sample I with J, K, and L shows that only L exhibits a leftward shift in the overall peak positions, indicative of MSI-high status. The light pink vertical bands indicate the positions of the reference peaks observed in wild-type mice

To focus on MSI at single loci, we conducted PCR-based fragment analysis using two established murine microsatellite markers, D3Mit10 (TG/AC repeat, *n* = 26) and mBat37 (A/T repeat, n = 37), in small intestinal tissues (Fig. 6). Wild-type mice, whether untreated or treated with KBrO₃ for four weeks, showed sharp and consistent peak profiles, indicating stable microsatellite lengths (Fig. 6A, B, G, H). In contrast, *Msh2*^*–/–*^ mice exhibited broadened peak distributions at both loci even without treatment, reflecting baseline MSI (Fig. 6C, I). Upon 4-week administration of KBrO₃, *Msh2*^*-/-*^ mice displayed further, although subtle, alterations in peak patterns (Fig. 6D, J), suggesting a slight enhancement of MSI under oxidative stress. In older *Msh2*^*–/–*^ mice (20 weeks of age), peak distributions were notably broader than in 10-week-old counterparts, indicating progressive accumulation of instability over time (Fig. 6E, K). Importantly, prolonged KBrO₃ treatment (16 weeks) in 20-week-old *Msh2*^*–/–*^ mice further exacerbated the peak diversity (Fig. 6F, L), implying a synergistic effect of age and oxidative stress in promoting MSI. Control and *Msh2*^*–/–*^ control mice at 10 weeks of age exhibited three consistent peaks.

### Driver mutations in Msh2^–/– ^tumors

We identified seven pathogenic driver mutations in either the *Apc* or *Ctnnb1* gene across six tumors analyzed, comprising both spontaneous and KBrO₃-induced cases (Table S3). All five mutations in *Apc* were frameshift mutations caused by 1–2 bp deletions in repeat sequences, leading to the loss of function of the APC protein. The two mutations in *Ctnnb1* were base substitutions that disrupted GSK3β phosphorylation sites. Such mutations in both genes are known to induce the constitutive activation of Wnt signaling, thereby promoting aberrant cellular proliferation. These findings suggest that the characteristic mutations detected in normal tissues prior to tumor formation using the *rpsL* assay play a direct role in tumor initiation.

## Discussion

Our study demonstrates that oxidative stress plays a pivotal role in promoting somatic mutagenesis and tumorigenesis in the MMR-deficient intestinal epithelium and further highlights the role of MSH2 as a key suppressor of oxidative stress–driven tumor development. Rather than acting as a classical tumor suppressor that immediately confers a selective growth advantage upon loss, MSH2 deficiency indirectly contributes to tumorigenesis by promoting a mutator phenotype that elevates the frequency of mutations in oncogenes and tumor suppressor genes, similar to that observed in MUTYH-deficient models [12]. Using the *rpsL* reporter gene assay, we identified a marked increase in somatic MF in normal tissues prior to tumor initiation upon chronic exposure to KBrO₃ in *Msh2*^*–/–*^ mice. Most mutations were single-base deletions within adenine mononucleotide repeats in the *rpsL* coding region, indicating that *rpsL*-based mutation profiling reflects genome-wide MSI. Mutation signature analysis revealed that signatures associated with MMR deficiency (SBS15, SBS44, ID2, and ID12) and clock-like processes (SBS1 and SBS5) were consistently detected across all *Msh2*^*–/–*^ tumors, similar to those observed in human MMR-deficient cancers. ID2, which involves 1-base deletions occurring in (A/T)_n_ tracts of six bases or longer, supports the findings of the *rpsL* assay. ID12 involves > 1 bp deletions occurring at repeat sites, although the source of the ID12 signature remains unclear. However, based on our MSI analysis using SE (Fig. 5), we propose that ID12 may represent the deletion of one to several units at dinucleotide repeat loci. In support of this notion, Owusu et al. have reported an association between ID12 and MMR deficiency in MMR-deficient human cancer cell lines [27].

Several mutational signatures have been linked to MMR deficiency, of which SBS15 and SBS44 were the most prominent in this analysis. One notable feature of SBS44 is the high frequency of C > T occurring in ACA motifs among the signatures associated with MMR deficiency. We found that C > T at ACA sites tended to be called at the junctions between mononucleotide repeats and non-repeat sequences. Therefore, it is likely that at least a subset of these mutations did not arise from a simple BS mechanism but rather resulted from multiple indel events occurring within regions containing simple tandem repeat sequences. Notably, the oxidative stress–associated signature SBS36 was additionally observed specifically in tumors derived from KBrO₃-treated mice, indicating that oxidative stress further aggravates the mutational landscape under MMR-deficient conditions. Fragment analysis also confirmed that KBrO₃ administration led to increased instability at canonical murine microsatellite loci. Importantly, driver mutations arise in tumor samples, particularly frameshift indels within the *Apc* gene and missense substitutions in *Ctnnb1*, both of which result in constitutive activation of the Wnt signaling pathway and promote cellular proliferation, supporting the hypothesis that oxidative stress not only increases the overall mutational burden but also contributes directly to oncogenic transformation in cryptic stem cells.

The *rpsL* assay proved highly effective for quantitatively assessing the impact of short-term oxidative stress exposure on repeat-site mutations in *Msh2*^*–/–*^ mice. These findings suggest that oxidative stress exacerbates the intrinsic instability of repetitive sequences in the absence of functional mismatch repair. While the *rpsL* assay enabled sensitive and quantitative detection of repeat-site mutations in normal tissues, WES/WGS analysis of tumors provided complementary genome-wide information on repeat instability at later stages of tumor development in *Msh2*^*–/–*^ mice. Instability was observed in mono- and dinucleotide repeats, including (A/T), (G/C), and (CA/TG) motifs, albeit to varying degrees. Because the MSI status varied depending on both the repeat motif and unit length, it was difficult to compare MSI levels between samples using a uniform metric such as the proportion of unstable loci. To evaluate genome-wide instability more comprehensively across repeat motifs, we applied SEanalysis to the WES/WGS data. Scatter plots of SE versus repeat unit numbers revealed distinct patterns between the MMR-proficient and MMR-deficient samples. SE quantifies allelic diversity at individual MS loci across different motifs and repeat lengths, reflecting variations in read length distributions. This was particularly effective in the mouse model with homozygous MMR gene deletions. The approach captures the “broadening of peak width” or “alteration of peak profiles” typically assessed in fragment analysis. Despite potential PCR artifacts inherent to WES data, SE analysis reliably distinguished MMR-deficient from MMR-proficient samples. SE increased with repeat length, even in MMR-proficient samples, suggesting intrinsic instability at certain MS loci. Moreover, we observed variable SE values, even among MS loci sharing the same motif and repeat length. These findings suggest that instability does not occur uniformly, even at loci with identical sequence profiles. Potential contributing factors include the surrounding genomic context, chromatin state, replication timing and direction, specific DNA polymerase involved in replication, and interactions with DNA damage and repair pathways.

In our previous study using the *gpt-*delta reporter gene system, no significant increase was observed in the overall MF in the small intestines of *Msh2*-deficient mice following KBrO₃ administration [21]. Although 1-bp deletions at (A/T)ₙ sites were detected, they did not increase the MF to a statistically significant level. One possible explanation for the discrepancy in the overall MF is that the target gene contains only short homopolymeric tracts, with the longest (A/T) repeat being five bases. In contrast, the *rpsL* reporter contains a 6-mer repeat, which is more prone to oxidative stress. Furthermore, it is plausible that the flanking sequence context surrounding the repeat regions influences their mutability, potentially modulating the frequency of indel formation in a sequence-specific manner. These findings suggest that mutation profiles, particularly in repeat sequences, vary depending on the reporter gene and assay system, which should be considered when assessing mutagenicity.

The molecular mechanism by which oxidative stress increases repeat instability has not been elucidated; however, we hypothesized that oxidatively damaged or modified DNA and nucleotides may compromise the stability of the DNA replication machinery. In support of this, our previous *rpsL* assay analyses revealed that mice deficient in *Mth1*(Nudt1) exhibited an increased frequency of 1-bp deletions in the (A)n tracts [19]. MTH1, encoded by the *Mth1* gene, is a nucleotide pool-sanitizing enzyme that prevents the incorporation of oxidatively damaged nucleotides during DNA replication, thereby reducing MF [28, 29]. Furthermore, *Mth1/Msh2* double-knockout mice exhibited a higher MF of 1-bp deletions at (A)ₙ tracts than either single-knockout model [19]. Russo et al. have demonstrated that oxidized deoxynucleoside triphosphates, particularly oxidized purines, such as 8-oxodGTP, contribute to mutagenesis in MMR-deficient cells. It has been shown that the overexpression of hMTH1 reduces spontaneous MF and MSI as well as the frequency of frameshift mutations at G/C and A/T mononucleotide repeats in MMR-defective human colorectal cancer cell lines [30]. These findings collectively suggest that an increased burden of oxidatively damaged nucleotides is also a source of replication errors that are normally corrected by the mismatch repair pathway and that these errors become more apparent under MMR-deficient conditions, thereby contributing to the instability observed in repeat sequences.

It has been reported that the interactions between the MMR and 8-oxoguanine repair via the BER pathway were previously suggested [31]. The gene expression of DNA polymerase beta, a key enzyme in the BER, is elevated in MSH2-deficient tumor cells compared with that in MSH2-proficient cells. Knockdown of DNA polymerase beta in MSH2-deficient human cancer cell lines induces synthetic lethality, which can be rescued by concurrent silencing of the *MUTYH* gene [31].

When oxidative stress occurs under MMR-deficient conditions, the accumulation of oxidative DNA damage, such as 8-oxoguanine, enhances BER pathway activity. However, excessive oxidative DNA lesions beyond the repair capacity of BER can lead to the accumulation of toxic repair intermediates, including DNA strand breaks, which are believed to trigger cell death. However, if MUTYH repair activity is compromised in MSH2-deficient cells under oxidative stress, the 8-oxoG:A mispair may remain unrepaired, leading to an increased frequency of G:C > T:A transversions. However, the absence of MUTYH-mediated repair may also prevent the formation of toxic repair intermediates, thereby preventing the activation of cell death pathways [32]. Therefore, these cells may gain a selective survival advantage under oxidative stress. However, considering that most driver gene mutations consist of G to A substitutions and frameshift mutations in repetitive sequences, which are both characteristic of MMR deficiency [20, 21], it is likely that MUTYH status did not play a major role in the initial acquisition of driver mutations in this model. Rather, reduced or lost MUTYH function may have contributed to enhancing the survival or clonal expansion of tumor cells under oxidative stress conditions, thereby playing a supportive role in tumor progression rather than initiation. This notion is further supported by the presence of oxidative stress–associated mutational signatures such as SBS36 in a wide range of human cancer genomes.

## Conclusions

Our findings underscore the pivotal role of oxidative stress in promoting MSI and somatic mutagenesis under MMR-deficient conditions. The *rpsL* reporter gene assay is a highly sensitive tool for detecting repeat-associated mutations that precede tumor development, revealing mutation patterns that are not readily captured using conventional sequencing or fragment-based approaches. These insights have significant implications for understanding how environmental and endogenous oxidative stress influence mutation accumulation and clonal expansion in precancerous tissues lacking functional mismatch repair. Importantly, this study demonstrated that MSH2 is essential for maintaining genome stability under oxidative conditions and acts as a key suppressor of oxidative stress–induced tumorigenesis. Future studies should further investigate the cooperative and antagonistic roles of specific DNA repair pathways in maintaining genome integrity under oxidative stress and how perturbations in these networks may create selective vulnerabilities or resistance mechanisms during tumor evolution.

## Supplementary Information

Additional file 1: Supplemental Fig. S1. Hotspots of adenine 1-indel mutations in the *rpsL* gene The mutation frequency (MF) of adenine 1-indel was calculated and mapped onto the *rpsL *coding sequence. The MF of deletion (left) and insertion (right) at each locus is indicated as deletion/insertion within colored panels. blue: control mice, bule: KBrO₃-treated mice. SD: Shine-Dalgarno sequence. Squares indicate start and stop codons. Mononucleotide adenine repeat sequences [(A)ₙ] are highlighted in magenta. The (A)₆ site emerged as the most frequent hotspot for adenine 1-indel mutations, followed by the (A)₅ site.Supplemental Fig. S2. Mutational profiles in *Msh2*^*-/-*^ tumor Mutational profiles based on tumor-specific variants identified using WES of tumors. Two tumors were isolated from a *Msh2*^*−/−*^ mouse treated with 0.15% KBrO₃. (A) SBS signature. (B) ID signature.

Additional file 2: Supplemental Table S1. *rpsL* assay mutatnt frequency.

Additional file 3: Supplemental Table S2. Tumor-specific mutations in tumor WGS.

Additional file 4: Supplemental Table S3. Driver mutation candidates.

Additional file 5: Supplemental Code.

## Data Availability

All data generated or analyzed during this study are included in this published article and its supplementary information files. The sequencing data have been deposited in the DDBJ under the BioProject accession number PRJDB37284.
