# Supplementary material for: Oxidative stress accelerates repeat sequence instability and base substitutions promoting gastrointestinal driver mutations in MSH2 deficient mice
Source: Genes Environ. 2025 Oct 23;47:19. doi: 10.1186/s41021-025-00342-y (PMC12548293; doi:10.1186/s41021-025-00342-y)
Supplement: Supplementary file 1 — Additional file 1: Supplemental Fig. S1. Hotspots of adenine 1-indel mutations in the rpsL gene The mutation frequency (MF) of adenine 1-indel was calculated and mapped onto the rpsL coding sequence. The MF of deletion (left) and insertion (right) at each locus is indicated as deletion/insertion within colored panels. blue: control mice, bule: KBrO₃-treated mice. SD: Shine-Dalgarno sequence. Squares indicate start and stop codons. Mononucleotide adenine repeat sequences [(A)ₙ] are highlighted in magenta. The (A)₆ site emerged as the most frequent hotspot for adenine 1-indel mutations, followed by the (A)₅ site.Supplemental Fig. S2. Mutational profiles in Msh2-/- tumor Mutational profiles based on tumor-specific variants identified using WES of tumors. Two tumors were isolated from a Msh2−/− mouse treated with 0.15% KBrO₃. (A) SBS signature. (B) ID signature. [file 41021_2025_342_MOESM1_ESM.pdf]

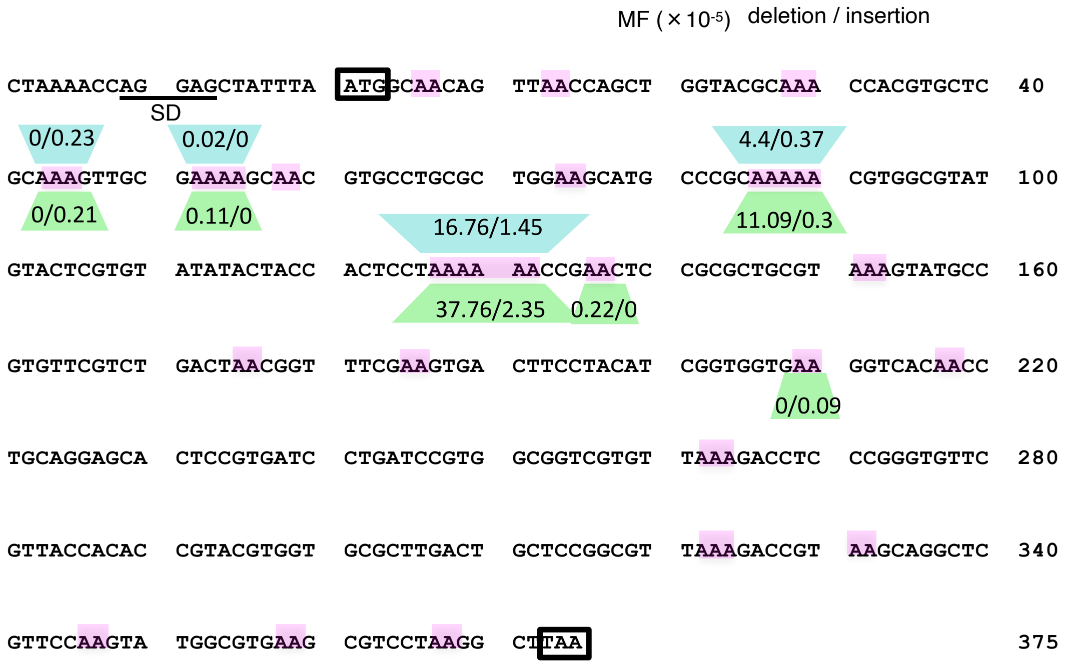

Supplemental Figure S1. Hotspots of adenine 1-indel mutations in the *rpsL* gene

The mutation frequency (MF) of adenine 1-indel was calculated and mapped onto the *rpsL* coding sequence. The MF of deletion (left) and insertion (right) at each locus is indicated as deletion/insertion within colored panels. blue: control mice, bule: $KBrO_3$-treated mice. SD: Shine-Dalgarno sequence. Squares indicate start and stop codons. Mononucleotide adenine repeat sequences [$(A)_n$] are highlighted in magenta. The $(A)_6$ site emerged as the most frequent hotspot for adenine 1-indel mutations, followed by the $(A)_5$ site.

A

B

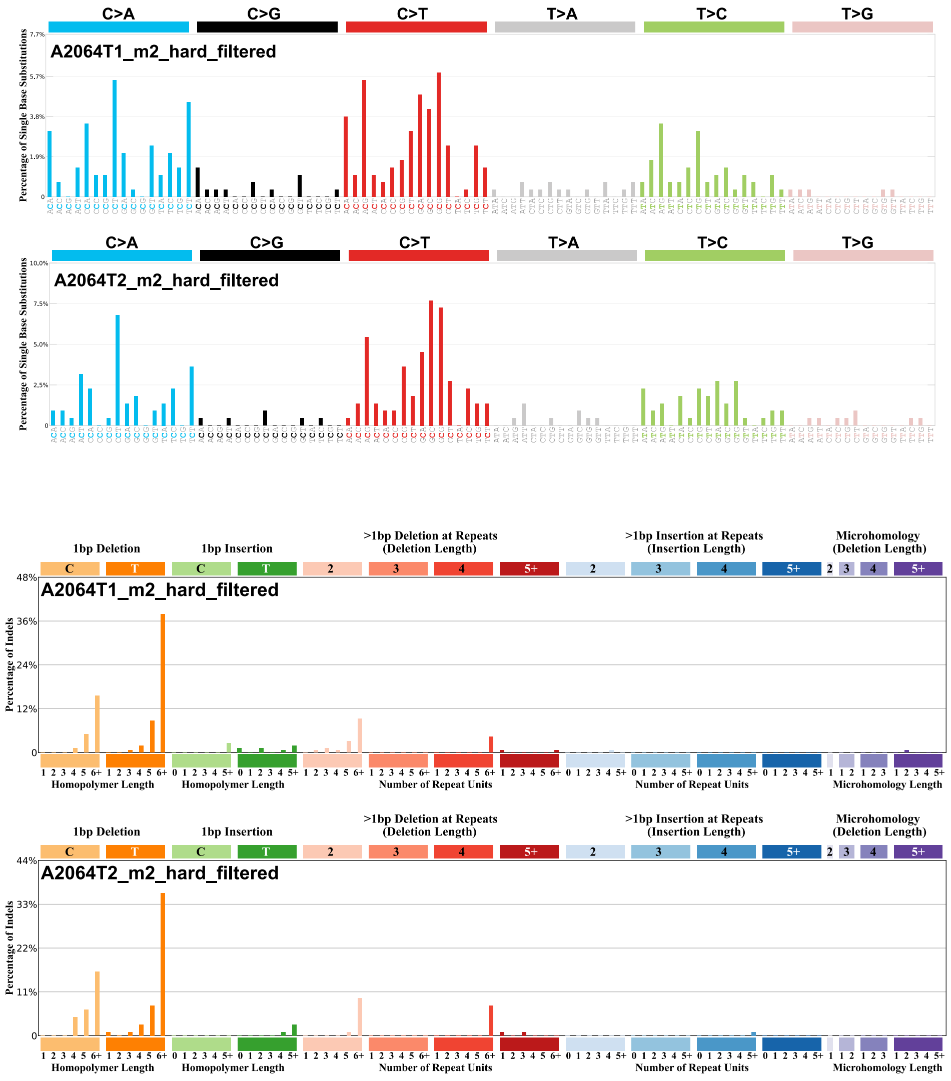

Supplemental Fig S2. Mutational profiles in *Msh2*$^{-/-}$ tumor

Mutational profiles based on tumor-specific variants identified using WES of tumors. Two tumors were isolated from a *Msh2*$^{-/-}$ mouse treated with 0.15% $KBrO_3$. (A) SBS signature. (B) ID signature.
